# CDKL2 promotes epithelial-mesenchymal transition and breast cancer progression

**DOI:** 10.18632/oncotarget.2535

**Published:** 2014-10-24

**Authors:** Linna Li, Chunping Liu, Robert J. Amato, Jeffrey T. Chang, Guangwei Du, Wenliang Li

**Affiliations:** ^1^ Texas Therapeutics Institute, Brown Foundation Institute of Molecular Medicine, University of Texas Health Science Center at Houston, Houston, Texas; ^2^ Division of Oncology, Department of Internal Medicine, and Memorial Hermann Cancer Center, University of Texas Health Science Center at Houston, Houston, Texas; ^3^ Department of Integrative Biology and Pharmacology, School of Medicine, University of Texas Health Science Center at Houston, Houston, Texas; ^4^ School of Biomedical Informatics, University of Texas Health Science Center at Houston, Houston, Texas; ^5^ Cancer Biology Program, Graduate School of Biomedical Sciences, University of Texas Health Science Center at Houston, Houston, Texas; ^6^ Department of Pharmacology and Toxicology, Beijing Institute of Radiation Medicine, Beijing, China

**Keywords:** cyclin-dependent kinase-like 2 (CDKL2), epithelial–mesenchymal transition (EMT), breast cancer, kinases, CD44

## Abstract

The epithelial–mesenchymal transition (EMT) confers mesenchymal properties on epithelial cells and has been closely associated with the acquisition of aggressive traits by epithelial cancer cells. To identify novel regulators of EMT, we carried out cDNA screens that covered 500 human kinases. Subsequent characterization of candidate kinases led us to uncover cyclin-dependent kinase-like 2 (CDKL2) as a novel potent promoter for EMT and breast cancer progression. CDKL2-expressing human mammary gland epithelial cells displayed enhanced mesenchymal traits and stem cell-like phenotypes, which was acquired through activating a ZEB1/E-cadherin/β-catenin positive feedback loop and regulating CD44 mRNA alternative splicing to promote conversion of CD24^high^ cells to CD44^high^ cells. Furthermore, CDKL2 enhanced primary tumor formation and metastasis in a breast cancer xenograft model. Notably, CDKL2 is expressed significantly higher in mesenchymal human breast cancer cell lines than in epithelial lines, and its over-expression/amplification in human breast cancers is associated with shorter disease-free survival. Taken together, our study uncovered a major role for CDKL2 in promoting EMT and breast cancer progression.

## INTRODUCTION

The ability of epithelial cells to undergo mesenchymal transitions during embryogenesis, wound healing and malignant progression is now widely accepted as a core biological process [1, 2]. Epithelial–mesenchymal transition (EMT) is characterized by loss of cell adhesion, repression of E-cadherin expression, increased cell mobility and expression of mesenchymal markers such as vimentin. During cancer progression, the process of EMT is associated with metastasis, therapeutic resistance, and stemness properties, which have been best demonstrated in breast cancer [3, 4].

The importance of EMT on cancer progression has been increasingly recognized over the last few years. Many regulators of EMT have been identified [1–4]. However, much of the work on EMT regulation is on a few upstream molecules (such as Wnt, Notch, Hedgehog, TGF-β and their receptors), several transcription factors (such as Twist, Snail, Slug, Zeb1, SIP1, FoxC2 and Six1) and microRNAs (such as microRNA-200 family members) [1–6]. Our knowledge in this critical process is still quite limited, especially in intracellular signaling pathways. Moreover, many of these known EMT regulators are traditionally considered as non-druggable or difficult to target.

Since little is known about the intracellular signaling pathways that lead to activation of EMT transcription factors, we focused on critical signaling molecules like kinases. Kinases play central roles in cell physiology and pathogenesis of human diseases, including cancer [7]. Kinase is also one of the most important gene families in cancer drug development [8]. Our and other's analyses indicated that the majority of human kinases have not been studied extensively [9–11]. In this study, we carried out a human cDNA library screen on 500 human kinases and identified a number of potential new EMT regulators. In particular, we uncovered that serine/ threonine kinase cyclin-dependent kinase-like 2 (CDKL2) is a potent promoter for EMT and breast cancer progression.

## RESULTS

### Kinase cDNA library screen for novel regulators of EMT

**Figure 1:**
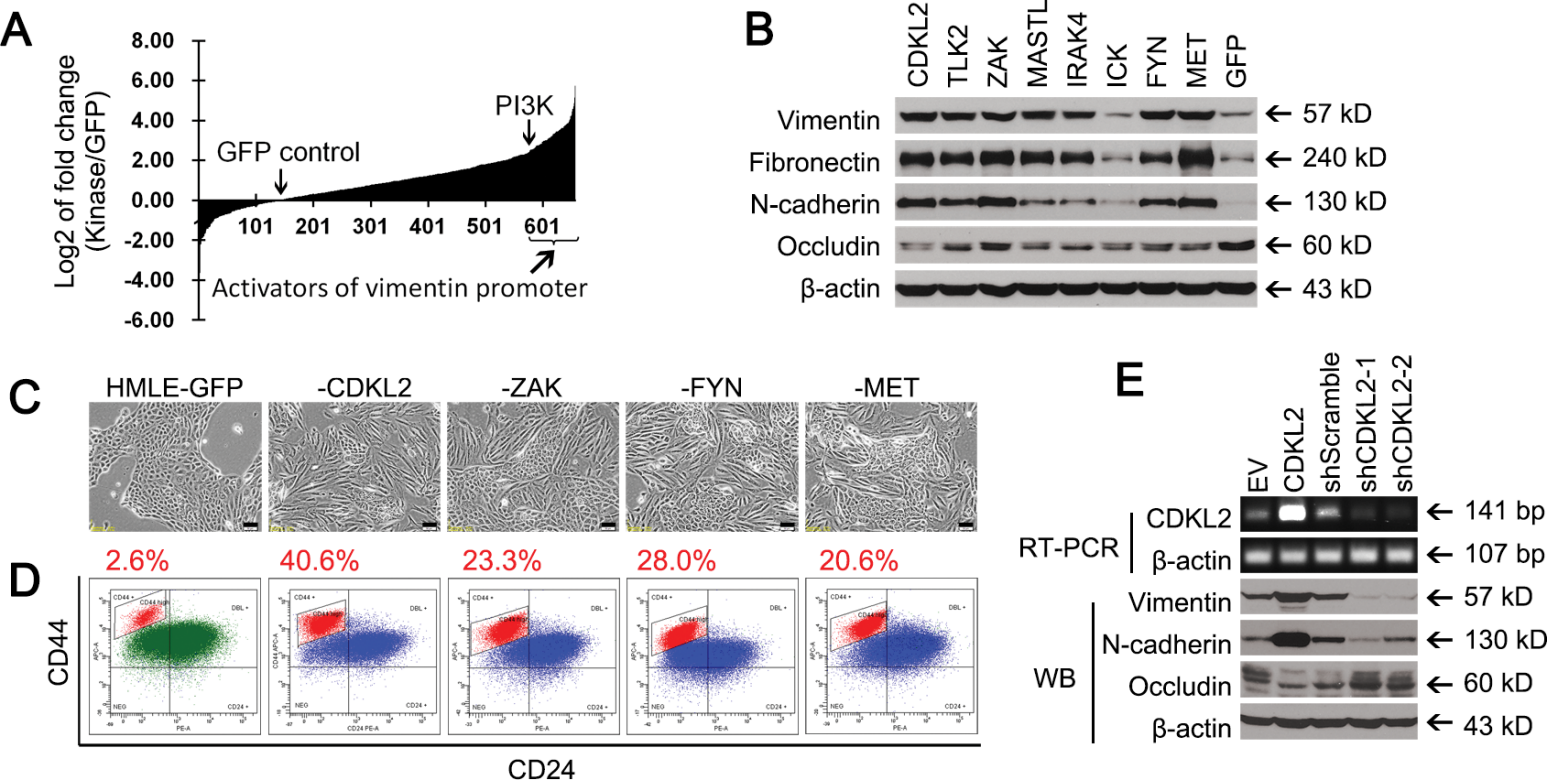
Human kinase cDNA screen and validation for novel regulators of EMT **(A)** human kinase cDNA screen was performed by measuring vimentin promoter luciferase reporter activities in firefly luciferase vector (VimPro-luc) that was transiently co-transfected with individual kinase cDNA vector and TK-renilla luciferase vector (pRL-TK, transfection control) into 293T cells. Shown on Y-axis is log2 transformation of the fold changes of vimentin promoter luciferase activity induced by individual kinases vs GFP control. X-axis represents 651 cDNA clones for 500 human kinases. **(B)** Western blotting analysis of mesenchymal markers (Vimentin, N-cadherin, and Fibronectin) and epithelial marker (Occludin) in stable HMLE cell lines expressing several candidate kinases, GFP negative control, and positive controls (MET and FYN). **(C)** mesenchymal-like morphological changes occurred in several stable HMLE cell lines expressing indicated kinases. Scale bars represent 50 μm. **(D)** FACS analysis of CD44 and CD24 in several stable cell lines. The percentage of the CD44^high^/CD24^low^ mesenchymal subpopulation is indicated. **(E)** shRNA silencing of CDKL2 gene led to downregulation of mesenchymal markers and upregulation of epithelial marker in HMLE cells, as measured by RT-PCR and western blotting analysis (EV, empty vector). A high quality antibody specific for CDKL2 is unavailable.

Since the induction of vimentin expression is one of the most prominent molecular hallmarks of EMT, we used a vimentin promoter (VimPro) [12] luciferase assay to identify potential EMT regulators. We surveyed a cDNA library consisting of 651 cDNA clones for 500 human kinases, and identified 55 kinases that activated VimPro better than PIK3CA, a known EMT promoter [15], with 7 fold induction of vimentin promoter activity relative to GFP control (Fig. 1A). Several kinases known to regulate EMT, stemness or metastasis, such as MET, FYN, PDGFRA, FLT1, AXL, BRAF, LYN and YES1 [16–19], were among the 55 potential EMT promoters. While this validated our screening approach, our main focus was to identify those kinase candidates that had not been implicated in EMT and cancer progression, and thus may be new targets for cancer therapy.

To validate and prioritize kinase candidates, we carried out several EMT *in vitro* cellular assays [1, 20, 21] using human mammary gland epithelial cells (HMLE), a classic EMT experimental model [13, 14, 22–26]. Consistent with results from the luciferase reporter assay in our cDNA screens, several under-studied kinase candidates and positive controls (FYN and MET) dramatically up-regulated the expression of mesenchymal markers, including vimentin, fibronectin and N-cadherin in HMLE cells (Fig. 1B). At the same time, down-regulation of epithelial marker occludin [21] was observed for some kinases (Fig. 1B). Besides changes in EMT marker expression, HMLE cells expressing some kinases, such as CDKL2, ZAK, FYN and MET, lost cell-cell contact and acquired a spindle, fibroblast-like mesenchymal morphology (Fig. 1C).

EMT has been associated with acquisition of stem cell-like properties, including expression of the putative breast cancer stem cell (CSC) marker CD44^high^/CD24^low^ [13, 27]. CDKL2, ZAK, FYN and MET promoted a 8–16 fold increase in the CD44^high^/CD24^low^ subpopulation in HMLE cells, compared to GFP control (Fig. 1D). Of note, among the kinase candidates, CDKL2-transduced cells demonstrated the most prominent EMT phenotypes, such as the most obvious mesenchymal morphology and the biggest increase in the CD44^high^/CD24^low^ subpopulation, better than positive controls FYN and MET. Therefore, CDKL2 was selected as our top candidate for further study.

In line with our objective to identify new regulators of EMT, very little is known about the function of CDKL2 in cellular physiology. Also known as p56 or KKIAMRE [28, 29], CDKL2 (cyclin-dependent kinase-like 2), is one of the most distant members of the cdc2-related serine/threonine protein kinase and mitogen-activated protein kinase (MAPK) family [29]. It was shown to be induced by EGF, suggesting that it may be involved in EGFR signaling [29]. It has also been shown to participate in learning and memory in mice [28, 30].

Since HMLE cells contain some CD44^high^/CD24^low^ mesenchymal cells and express measureable levels of mesenchymal markers vimentin and N-cadherin, we next examined whether the detectable mesenchymal phenotypes could be weakened through shRNA-mediated down-regulation of CDKL2 gene. Compared to Scramble shRNA control, two CDKL2 shRNA-1 and -2 targeting different regions of CDKL2 mRNA clearly decreased CDKL2 gene expression in HMLE cells, and induced opposite patterns of EMT marker expression as compared to CDKL2 cDNA in HMLE cells (Fig. 1E). These cDNA and shRNA results suggest that CDKL2 plays a critical role in EMT in HMLE cells, for which we employed the following studies for further validation.

### CDKL2 is a novel promoter for EMT and stem cell-like phenotypes

**Figure 2:**
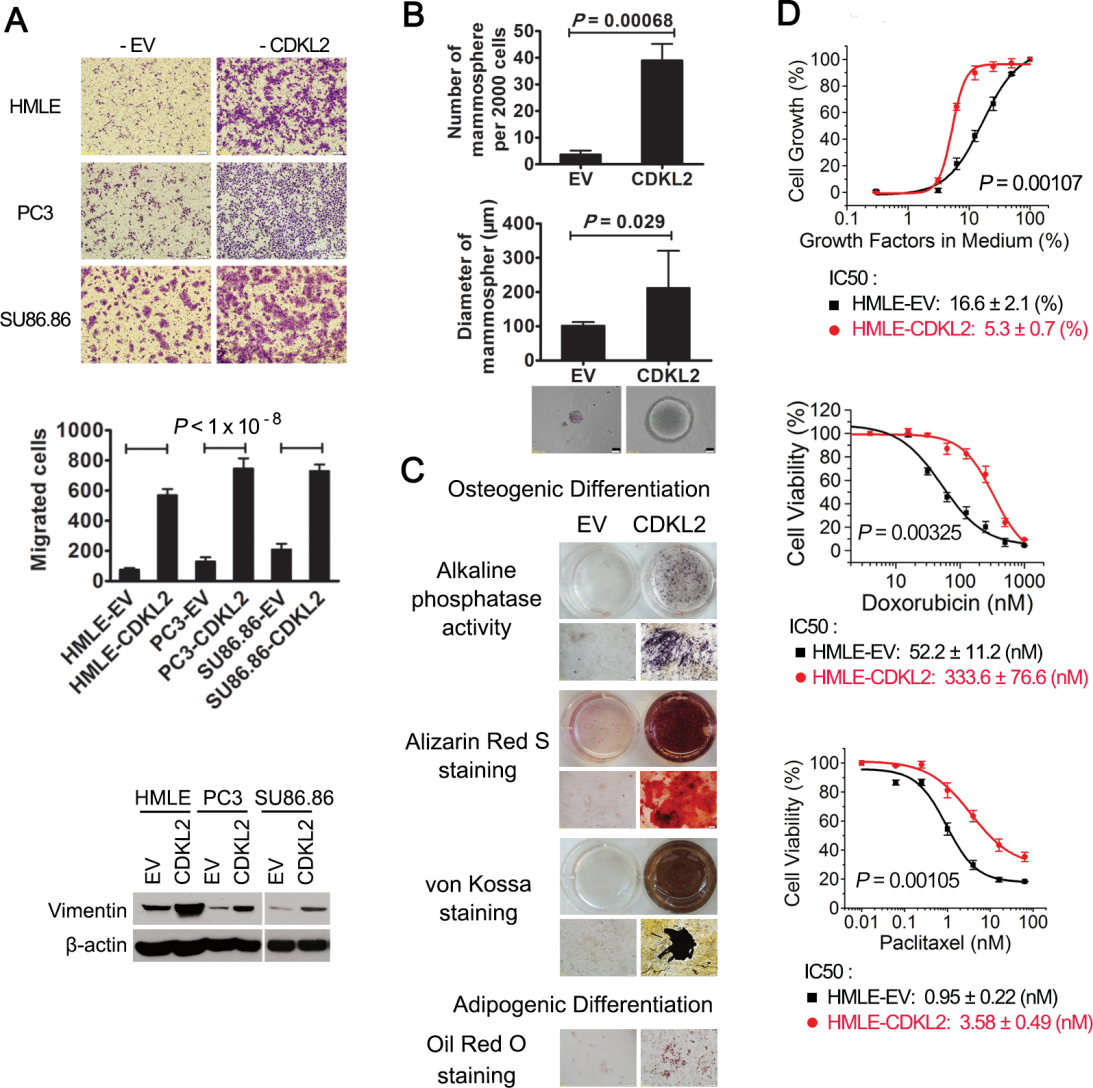
CDKL2-transduced cells show both EMT and stem cell-like phenotypes **(A)** CDKL2 induced migration and EMT marker expression in 3 epithelial cell lines, showing representative photos of migration (top), quantification of migration as the mean ± SD (middle) and vimentin expression (bottom). **(B)** HMLE-CDKL2 cells generated more mammospheres than HMLE-EV control cells. Phase-contrast images represent mammospheres formed by indicated cell lines. **(C)** HMLE cells expressing CDKL2 gained MSC-like capabilities for multilineage differentiation. Following culture in osteoblastic differentiation media, cells were tested for alkaline phosphatase (AP) activity, or analyzed by alizarin red S staining and silver nitrate (Von-Kossa) staining to determine calcium deposition and mineral deposition. Following culture in adipogenic differentiation media, cells were stained with oil red dye to detect oil droplets formation. **(D)** dose-response in survival and proliferation of HMLE-EV and HMLE-CDKL2 cells treated with different concentrations of paclitaxel or doxorubicin, or incubated with reducing concentrations of growth factors. IC50 values were obtained by using logistic nonlinear regression analyzing model of MicroCal Origin 7.0 software. Error bars denote SD from quadruplicate.

Increased migration is a classic feature of EMT cells. As expected, HMLE-CDKL2 showed higher migratory ability than vector control cells in Boyden chamber assay (Fig. 2A). Since EMT has also been found in epithelial cell of other tissue types, we suspected that CDKL2 plays a similar role in other epithelial cells. Indeed, prostate cancer epithelial cell line PC3 and pancreatic cancer epithelial cell line SU86.86 showed increased vimentin expression and enhanced migration ability with CDKL2 ectopic expression (Fig. 2A). These results suggest that CDKL2's role on EMT regulation is not limited to HMLE mammary gland epithelial cells.

As a further validation for its role in promoting stem cell-like phenotypes, CDKL2 induced a clear increase in the ability to form mammosphere, an *in vitro* measure of stemness [31] (~8 fold increase in sphere numbers and ~2 fold increase in sphere diameters), relative to vector control cells (Fig. 2B). Its capability of promoting mammosphere formation was comparable to that of Snail and Twist, two well-documented EMT promoters [13] (Supplementary Fig. S1A). Furthermore, we evaluated stemness of HMLE-CDKL2 cells by examining their multilineage differentiation ability. CDKL2 overexpression was capable of inducing HMLE cells to undergo osteoblast and adipocyte differentiation in corresponding differentiation media (Fig. 2C), similar to Snail- and Twist-induced EMT cells and human mesenchymal stem cells [14](Supplementary Fig. S1B). These evidences suggest that CDKL2 gene not only promotes HMLE cells undergoing EMT but also enhances their stem cell-like characteristics, such as self-renewal ability in suspension culture and potentials for multilineage differentiation.

Cells that have undergone EMT have also been shown to be resistant to many hostile factors including nutrition deprivation and anticancer treatment [32, 33]. When grown in growth factors reduced media, HMLE-CDKL2 cells showed a more robust proliferative capacity than control cells (Fig. 2D). Furthermore, HMLE-CDKL2 cells were more resistant to two commonly used chemotherapeutic drugs, paclitaxel and doxorubicin, with ~4-fold and 6-fold increase in IC50, respectively, as compared with the vector control cells (Fig. 2D). Together, these results establish an important role in EMT and stem cell-like phenotypes for CDKL2, a kinase with previously unknown function.

### CDKL2 endows CD44^high^/CD24^low^ mesenchymal subpopulation with enhanced EMT and stem cell phenotypes

**Figure 3:**
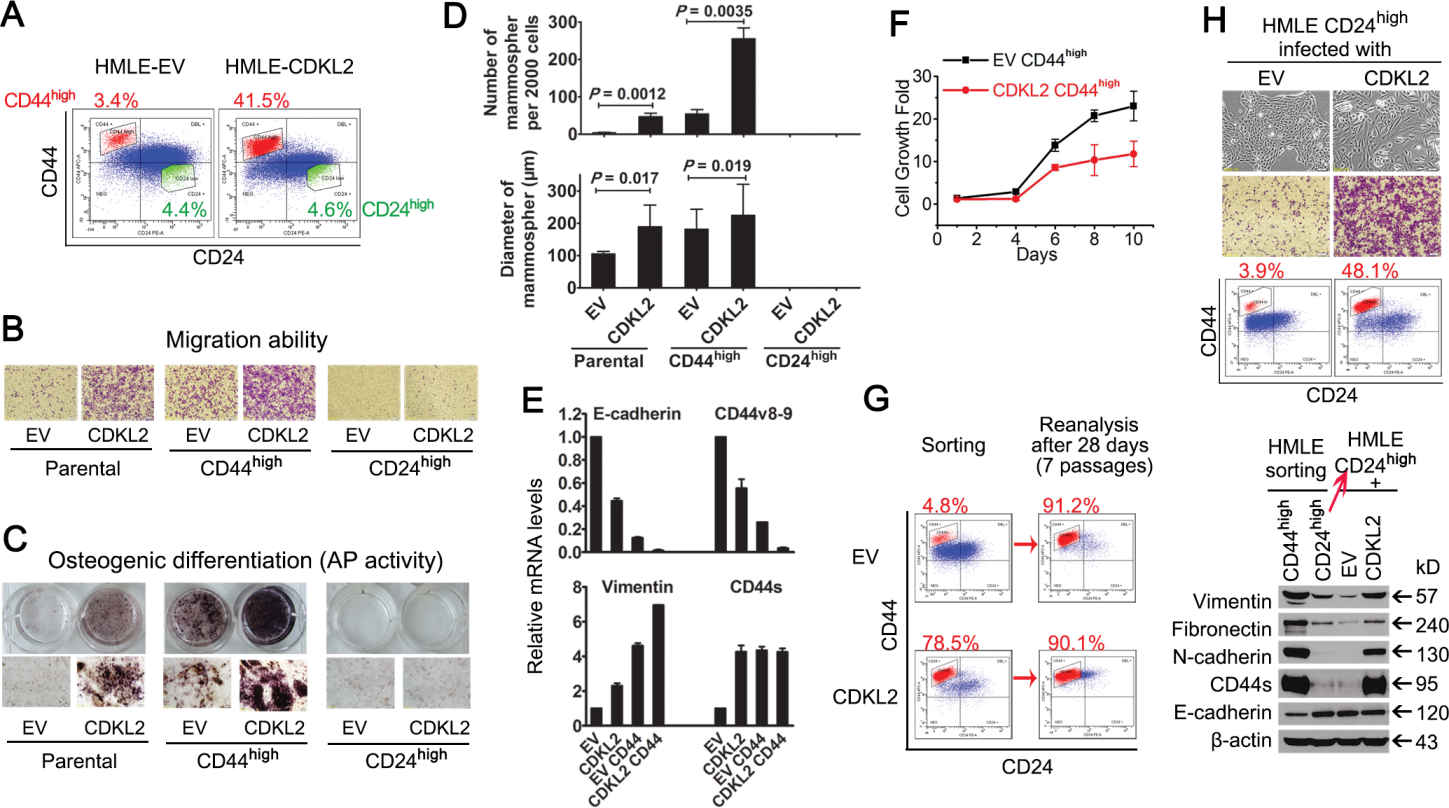
CDKL2 promotes direct transition of CD24^high^ epithelial cells into CD44^high^ cells and endows them with enhanced EMT and stem cell-like phenotypes **(A)** CD44^high^ (short for CD44^high^/CD24^low^) and CD24^high^ (short for CD44^low^/CD24^high^) subpopulations of HMLE-EV and HMLE-CDKL2 parental cells were sorted out by FACS. The percentage of sorted cells is indicated. **(B)** representative photos of migration of above cells determined by Boyden Chamber assay. **(C)** osteogenic differentiation ability (Alkaline Phosphatase activity) of parental and sorted cells. **(D)** mammospheres formed by parental and sorted cells. **(E)** relative expression of epithelial markers (E-cadherin and CD44v8-9) and mesenchymal markers (Vimentin and CD44s) was measured by qRT-PCR. The data are reported as mean ± SD. **(F)** proliferation curves of CD44^high^ sorted cells indicated that CD44^high^ subpopulation from HMLE-CDKL2 cells did not proliferate faster than those from HMLE-EV cells. Bars denote standard error from quadruplicate. **(G)** FACS re-analysis after 7 passages of continuous culture of CD44^high^ sorted cells indicated that most CD44^high^ cells from HMLE-EV and -CDKL2 cells remained CD44^high^. **(H)** EV and CDKL2 were re-introduced into CD24^high^ epithelial subpopulation sorted from HMLE parental cells. After 4 weeks of drug selection and *in vitro* passaging, cell migration ability, CD44/CD24 and EMT markers expression were re-analyzed. These results indicated that increased CD44^high^ subpopulation in HMLE-CDKL2 cells was a result of true EMT by CDKL2 converting CD24^high^ cells into CD44^high^ cells.

Since CD44^high^/CD24^low^ mesenchymal cells have been linked to increased migration ability and stem cell characteristics in breast cancer [13, 14, 25], we reasoned that the HMLE-CDKL2 phenotypes we observed might merely be due to an increase in the percentage of the CD44^high^/CD24^low^ subpopulation. To test this, we sorted out CD44^high^/CD24^low^ mesenchymal cells (referred as CD44^high^ hereafter) and CD24^high^/CD44^low^ epithelial cells (referred as CD24^high^ hereafter) from both HMLE-EV and HMLE-CDKL2 cells (Fig. 3A). Surprisingly, the CD44^high^ subpopulation sorted from HMLE-CDKL2 cells clearly showed higher capabilities in migration, mammosphere formation and multilineage differentiation than the CD44^high^ subpopulation sorted from HMLE-EV cells, although they were both CD44^high^ and with mesenchymal morphology (Fig. 3B-D and Supplementary Fig. S2A-C). Epithelial CD24^high^ subpopulations of both cell lines were incapable of migrating, sphere-forming and differentiating under the same conditions. qRT-PCR analysis further revealed that epithelial markers E-cadherin, CD24 and CD44v8-9 (epithelial isoform of CD44) showed even more decreases (6~14 fold) in HMLE-CDKL2-CD44^high^ cells than in HMLE-EV-CD44^high^ cells, while mesenchymal marker vimentin exhibited an opposite expression pattern (Fig.3E and Supplementary Fig. S2B). These results were consistent with previous reports showing that CD44^high^ cells are not homogeneous in EMT and stem cell-like characteristics [23, 34], and indicate that CDKL2 could largely enhance these characteristics in CD44^high^ of HMLE cells.

### CDKL2 promotes direct transition of CD24^high^ epithelial cells into CD44^high^ mesenchymal cells

Next, we set out to determine how CDKL2 increased the proportion of CD44^high^ mesenchymal subpopulation in HMLE cells. We considered that there might be three equally interesting possibilities. Firstly, CDKL2 could promote proliferation of CD44^high^ subpopulation and thus increase its proportion in a population. Secondly, CDKL2 could inhibit the differentiation of stem cell-like CD44^high^ cells into epithelial CD24^high^ cells and thus reduce the proportion of CD24^high^ subpopulation in a population. Thirdly, CDKL2 could promote direct transition from CD24^high^ epithelial cells to CD44^high^ mesenchymal cells, which is a true EMT process.

Firstly, we found that there was no proliferation advantage for HMLE-CDKL2-CD44^high^ cells. Instead, they grew slower than HMLE-EV-CD44^high^ cells (Fig. 3F), so we ruled out the first possibility. Secondly, about 90% of CD44^high^ subpopulations from either HMLE-CDKL2 or HMLE-EV cells still maintained their original CD44/CD24 phenotypes after continuous culture for 28 days (7 passages) (Fig. 3G), and no significant CD24^high^ subpopulation emerged, indicating that there was no significant difference in differentiation ability from the CD44^high^ subpopulation into the CD24^high^ subpopulation between HMLE-CDKL2 and HMLE-EV cells. Therefore, the second possibility was also ruled out.

To test the third possibility that CD44^high^ cells directly transited from CD24^high^ cells upon CDKL2 expression, we introduced either empty vector (EV) or CDKL2 into the CD24^high^ epithelial subpopulation of HMLE cells. EV-transduced CD24^high^ cells remained irregular islands of highly compact epithelial cells, while many mesenchymal cells appeared in CDKL2-transduced CD24^high^ cells (Fig. 3H). Consistently, a 10-fold increase of CD44^high^ subpopulation appeared in CDKL2-transduced CD24^high^ cells (Fig. 3H). A strong mesenchymal expression pattern was also found in CDKL2-transduced CD24^high^ cells. Collectively, these results indicate that CDKL2 could promote EMT through inducing the direct transition from CD24^high^ epithelial cells to CD44^high^ mesenchymal cells.

### CDKL2 promotes EMT and increases CD44^high^ subpopulation through up-regulating ZEB1 expression

**Figure 4:**
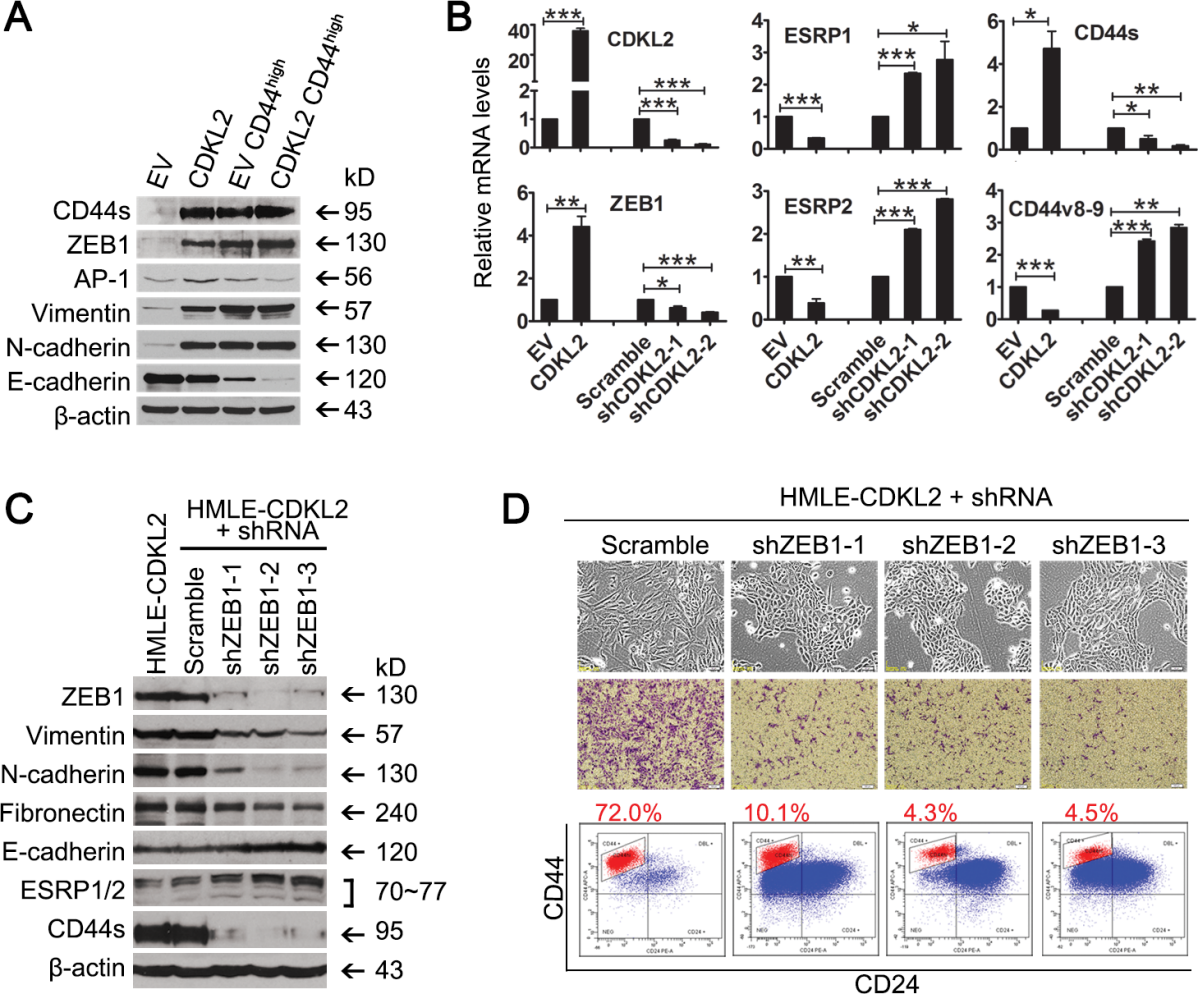
ZEB1 is a key mediator in CDKL2-induced EMT **(A)** Western blotting analysis of EMT markers (E-cadherin, Vimentin, N-cadherin, Fibronectin), EMT regulators (ZEB1 and AP1) in parental and CD44^high^ subpopulation of HMLE-EV and HMLE-CDKL2 cells. **(B)** changes in the expression of ZEB1, ESRP1/2, CD44s and CD44v8-9 by CDKL2 knockdown in HMLE cells as determined by real time PCR. *P<0.05, **P<0.01, ***P<0.001. C and D, shRNA silencing of ZEB1 gene and resultant reversal of EMT in HMLE-CDKL2 cells. Showed are changes in the expression of ZEB1, EMT markers, ESRP1/2 and CD44s **(C)** as well as cellular morphology, migration ability and CD44/CD24 antigenic profile **(D)** by ZEB1 knockdown in HMLE-CDKL2 cells.

To investigate the mechanism underlying CDKL2 promoting EMT and augmenting CD44^high^ mesenchymal cells, we turned to examine transcription factors known to be critical for EMT [1–4, 21]. We found that mRNA levels of ZEB1, ZEB2, FOXC2 and AP1 were expressed higher in parental HMLE-CDKL2 cells than in parental HMLE-EV cells, while Twist, Snail and Slug were not (Supplementary Fig. S3). Among those four transcription factors that were elevated in parental HMLE-CDKL2 cells, only ZEB1 mRNA and protein were further up-regulated in HMLE-CDKL2-CD44^high^ cells (Fig. 4A and Supplementary Fig. S3), coinciding with the enhanced EMT and stem cell-like characteristics of HMLE-CDKL2-CD44^high^ cells. Therefore, we focused on ZEB1 up-regulation for our CDKL2 mechanistic investigation.

Given that ZEB1 could repress the expression of ESRPs (epithelial splicing regulatory proteins) [21, 35] that regulate CD44 alternative splicing during EMT [22, 26], we tested whether ZEB1 regulated ESRPs expression in HMLE cells with CDKL2 overexpression or knockdown. Indeed, up-regulation of ZEB1 and decreased expression of ESRP1/2 were detected in HMLE-CDKL2 cells (Fig. 4B). The switch in CD44 isoforms expression (up-regulation of mesenchymal isoform CD44s and down-regulation of epithelial isoform CD44v8-9) was also evidenced (Fig. 4B). In contrast, when CDKL2 expression was inhibited by effective shRNAs, opposite changes in expression of ZEB1, ESRP1/2, and CD44 isoforms were observed (Fig. 4B). These results indicate that, during CDKL2-induced EMT, ZEB1 could repress ESRP1/2 expression, which led to a shift of CD44 expression from epithelial isoforms (CD44v8-9) to mesenchymal isoform (CD44s).

Consistent with a critical role of ZEB1 in CDKL2-induced increase of CD44^high^ subpopulation, decreased expression of mesenchymal markers (vimentin, fibronectin and N-cadherin) and an increased expression of E-cadherin were found in HMLE-CDKL2 cells expressing three ZEB1 shRNAs that effectively silenced ZEB1 expression (Fig. 4C). These cells exhibited strong ESRP1/2 expression, weak CD44s expression, loss of their fibroblast-like mesenchymal morphology and restored epithelial morphology of tight adhering cells in irregular islands, as compared to HMLE-CDKL2-shScramble cells (Fig. 4C and D). Functionally, *in vitro* migration ability of HMLE-CDKL2 cells was significantly decreased when ZEB1 expression was reduced (Fig. 4D). These results further confirmed the reversal of EMT caused by ZEB1 suppression in HMLE-CDKL2 cells. Furthermore, HMLE-CDKL2 cells expressing effective ZEB1 shRNAs exhibited 7-17 fold decreases in the proportion of CD44^high^ subpopulation as compared with Scramble shRNAcontrol (Fig. 4D). Taken together, ZEB1 is a key mediator in CDKL2-induced EMT and enhances CD44^high^ mesenchymal phenotypes.

### CDKL2 activates a positive feedback loop composed of ZEB1, E-cadherin and β-catenin

**Figure 5:**
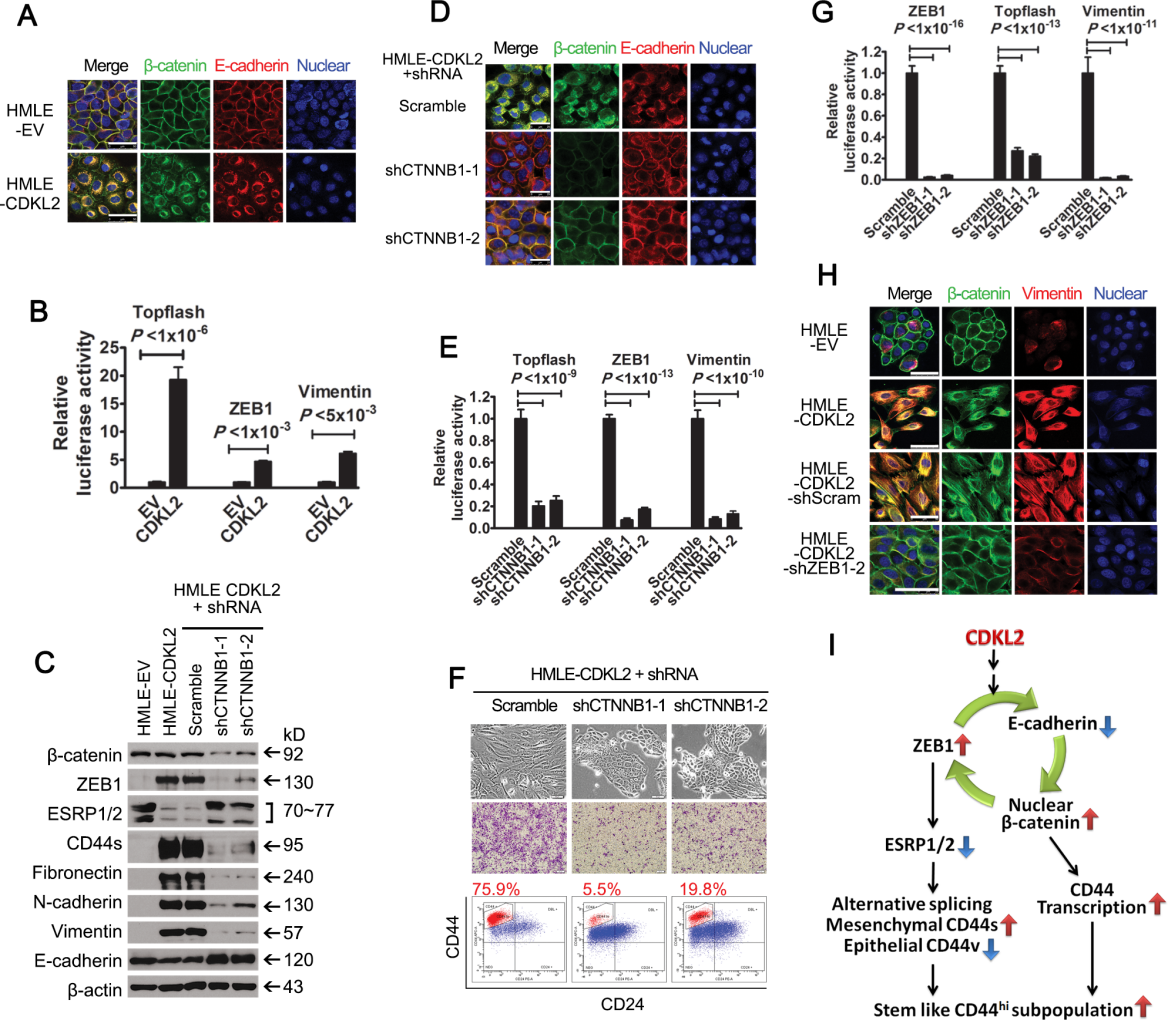
CDKL2 activates a positive feedback loop composed of ZEB1, E-cadherin and β-catenin **(A)** immunofluorescence images of HMLE-EV and -CDKL2 cells stained for β-catenin and E-cadherin. Cell nuclei were stained with DRAQ5. Scale bars, 50 μm. **(B)** luciferase activity of TOPflash, ZEB1 and vimentin promoter luciferase-reporter constructs in HMLE-EV and -CDKL2 cells. (C-F) breaking the loop by β-catenin (*CTNNB1*) silencing in HMLE-CDKL2 cells resulted in reversal of CDKL2-induced EMT. Shown are alterations in the expression of ZEB1, EMT markers, ESRP1/2 and CD44s **(C)**, immunofluorescence staining patterns of β-catenin and E-cadherin **(D)**, luciferase reporter activities of TOPflash, ZEB1 and vimentin promoters **(E)**, as well as morphology, CD44/CD24 antigenic profile and migration ability **(F)**. Scale bar, 50 μm. (G-H) breaking the loop by ZEB1 silencing in HMLE-CDKL2 cells. Shown are alterations in luciferase reporter activities of TOPflash, ZEB1 and vimentin promoters **(G)**, as well as the immunofluorescence staining patterns of β-catenin and vimentin **(H)**. Scale bars, 25 μm. **(I)** a proposed model of CDKL2 regulation on CD44^high^ and stem cell-like phenotypes through activation of a positive feedback loop composed of ZEB1, E-cadherin and β-catenin. The loop, on one hand, promotes CD44 transcription through activating Wnt/β-catenin signaling; on the other hand, represses ESRP1/2 expression by upregulating ZEB1, which promotes CD44 alternative splicing, resulting in a switch in expression from epithelial CD44v isoforms to mesenchymal CD44s isoform and CD44^high^ subpopulation increases over the time not only in quantity but also with enhanced EMT and stem cell-like phenotypes.

We wished to further investigate the mechanism of EMT induced by CDKL2 and ZEB1. ZEB1 has been identified as an effector of Wnt/β-catenin signaling [36] and a transcriptional repressor of the E-cadherin (CDH1) gene [37]. E-cadherin plays dual roles in epithelial cells: a cell-cell adhesion molecule and a negative regulator of the canonical Wnt signaling cascade [38]. We have showed increased expression of ZEB1 and decreased expression of E-cadherin in CDKL2 overexpressed cells (Fig. 3E, 4A and Supplementary Fig. S2B), which suggested that breakdown of cadherin/catenin-based adhesion system and activation of Wnt/β-catenin signaling may occur in HMLE-CDKL2 cells. In HMLE-EV cells, β-catenin was predominantly at the membrane, where it co-localized with E-cadherin (Fig. 5A). In contrast, an obvious translocation of both β-catenin and E-cadherin from membrane to perinuclear region was observed in HMLE-CDKL2 cells (Fig. 5A). We next examined if these changes also lead to the activation of the Wnt pathway. Indeed, a ~20-fold increase of TCF4 promoter activity, a readout for Wnt pathway activation, and a ~5-fold increase of ZEB1 promoter activity were detected in HMLE-CDKL2 cells, as compared to vector control cells (Fig. 5B), which suggest a positive feedback loop composed of ZEB1, E-cadherin and β-catenin is activated in HMLE-CDKL2 cells.

We then set out to determine whether EMT induced by CDKL2 can be reversed by breaking the positive feedback loop. Indeed, effective reduction of β-catenin expression and β-catenin/TCF4 transcriptional activity by β-catenin (CTNNB1) shRNAs in HMLE-CDKL2 cells led to decreased ZEB1 expression level and promoter activity, as well as increased E-cadherin expression and redistribution from perinuclear region to membrane (Fig. 5C-E). As a result, decreased expression of mesenchymal markers and CD44s, reduced transcriptional activity of the vimentin promoter, along with more epithelial morphology, reduced CD44^high^ subpopulations and migration ability were observed in HMLE-CDKL2 with β-catenin knockdown (Fig. 5C-F). This is consistent with an earlier report where β-catenin silencing reversed EMT induced by CDH1 down-regulation [39]. We further tested if a breakdown of the positive feedback loop could also be obtained by ZEB1 silencing. Certainly, several effective ZEB1 shRNAs can reverse the EMT phenotypes by CDKL2 overexpression, similar to β-catenin knockdown (Fig. 4C, 4D, 5G, 5H, Supplementary Fig. S4). Therefore, the positive feedback loop composed of ZEB1, E-cadherin and β-catenin, as depicted in Fig.5I, plays a key role in EMT induced by CDKL2.

Meanwhile, since activated ZEB1 repressed ESRP1/2 expression that promoted a switch in expression from epithelial CD44v8-9 isoform to mesenchymal CD44s isoform [22, 26], it was not surprising to observe that the proportion of CD44^high^ subpopulation in HMLE-CDKL2 cells increased over the time, as a result of the positive feedback loop. The freshly established HMLE-CDKL2 stable cell lines (in about 4 weeks) contained 40~50% of CD44^high^ subpopulation (Fig. 1D, 3A, 3H). Along with the increased culturing time, the proportion of CD44^high^ subpopulation was also increased. After about 3 months, CD44^high^ subpopulation in HMLE-CDKL2 cells increased to 70~80% (Fig. 3G, 4D, 5F).

### CDKL2 promotes tumor formation and metastasis

**Figure 6:**
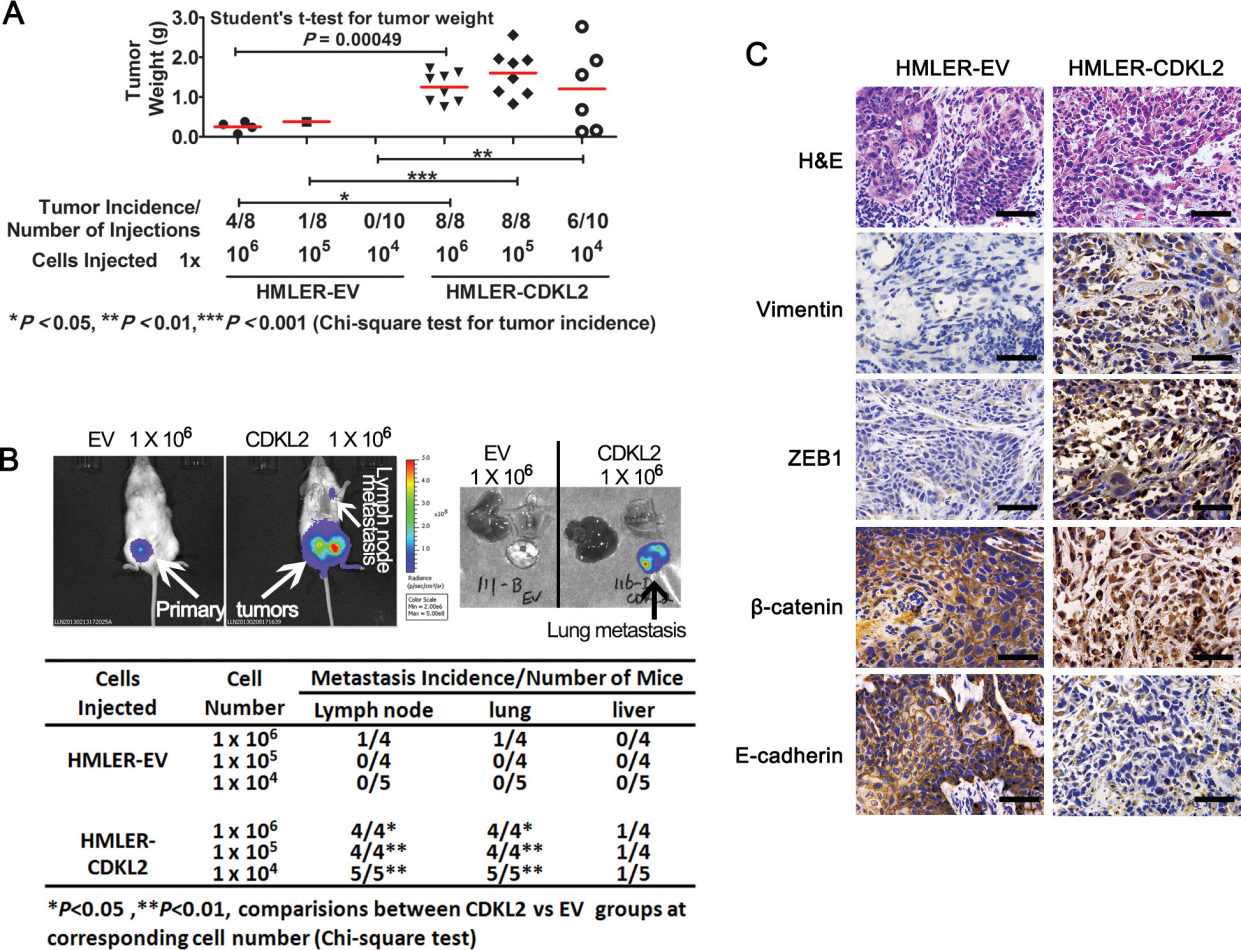
CDKL2 promotes mammary gland tumor formation and lung metastasis in orthotopic xenograft mouse model **(A)** tumor incidence and tumor weight of transformed HMLER cells expressing CDKL2 or EV. **(B)** *in vivo* and *ex vivo* bioluminescence imagining of NOD/SCID mouse hosts injected with HMLER cells expressing either EV or CDKL2 into the fourth inguinal mammary glands. The fourth inguinal mammary glands on either side of the same mice were injected with the same cell lines. Shown on left panel are representative *in vivo* images of orthotopic tumors and lymph node metastases 5 weeks after inoculation. Shown on right panel is representative *ex vivo* bioluminescence images of lung, liver and chest bones. **(C)** histological analysis of tumors developed from HMLER-EV and -CDKL2 cells. Shown are H&E and IHC staining for β-catenin, ZEB1, vimentin and E-cadherin. Scale bars, 200 μm.

In order to compare the tumorigenic and metastatic potential of CDKL2 and EV expressing cells in mice, HMLER cells (HMLE cells transformed with H-RasV12 oncogene that renders them tumorigenic) expressing either EV or CDKL2 were injected into the inguinal mammary glands of immunodeficient NOD/SCID mice in limiting dilutions. Six of ten injections with 1×10^4^ HMLER-CDKL2 cells generated primary tumors, while no tumors arose when an equal number of HMLER-EV cells were injected into mice (Fig. 6A). All injections with 1×10^5^ (8/8) or 1×10^6^ (8/8) HMLER-CDKL2 cells formed primary tumors, while same numbers of HMLER-EV cells generated primary tumors with lower frequency (1/10 for 1×10^5^, and 4/8 for 1×10^6^) and reduced size (5–6 times smaller on average) (Fig. 6A). Therefore, expression of CDKL2 gene significantly increased tumor incidence of HMLER cells.

Furthermore, *in vivo* and *ex vivo* bioluminescence imagining was used to detect metastasis in mouse hosts (Fig. 6B). Lymph node and lung metastases were observed in all the mice injected with HMLER-CDKL2 cells (Fig. 6B). Some mice with HMLER-CDKL2 tumors also showed liver metastases. However, only one mouse from HMLER-EV group had metastases in lymph nodes and lung. Hence, expression of CDKL2 significantly enhanced spontaneous metastasis.

The histology of tumors developed from HMLER-EV cells appeared as squamous metaplasia with nesting arrangement, while HMLER-CDKL2 tumors displayed a diffuse arrangement, absence of tumor nests and cord-like supporting structure (Fig. 6C). Furthermore, the EV tumors showed membrane staining of E-cadherin and β-catenin, while HMLER-CDKL2 tumors showed nuclear and cytoplasmic β-catenin staining and loss of E-cadherin staining, and significantly increased expressions of CDKL2, ZEB1 and vimentin (Fig. 6C). These results indicate that orthotopic xenograft mammary gland tumors generated by HMLER-CDKL2 cells maintain mesenchymal phenotypes.

### CDKL2 expression in human breast cancers

**Figure 7:**
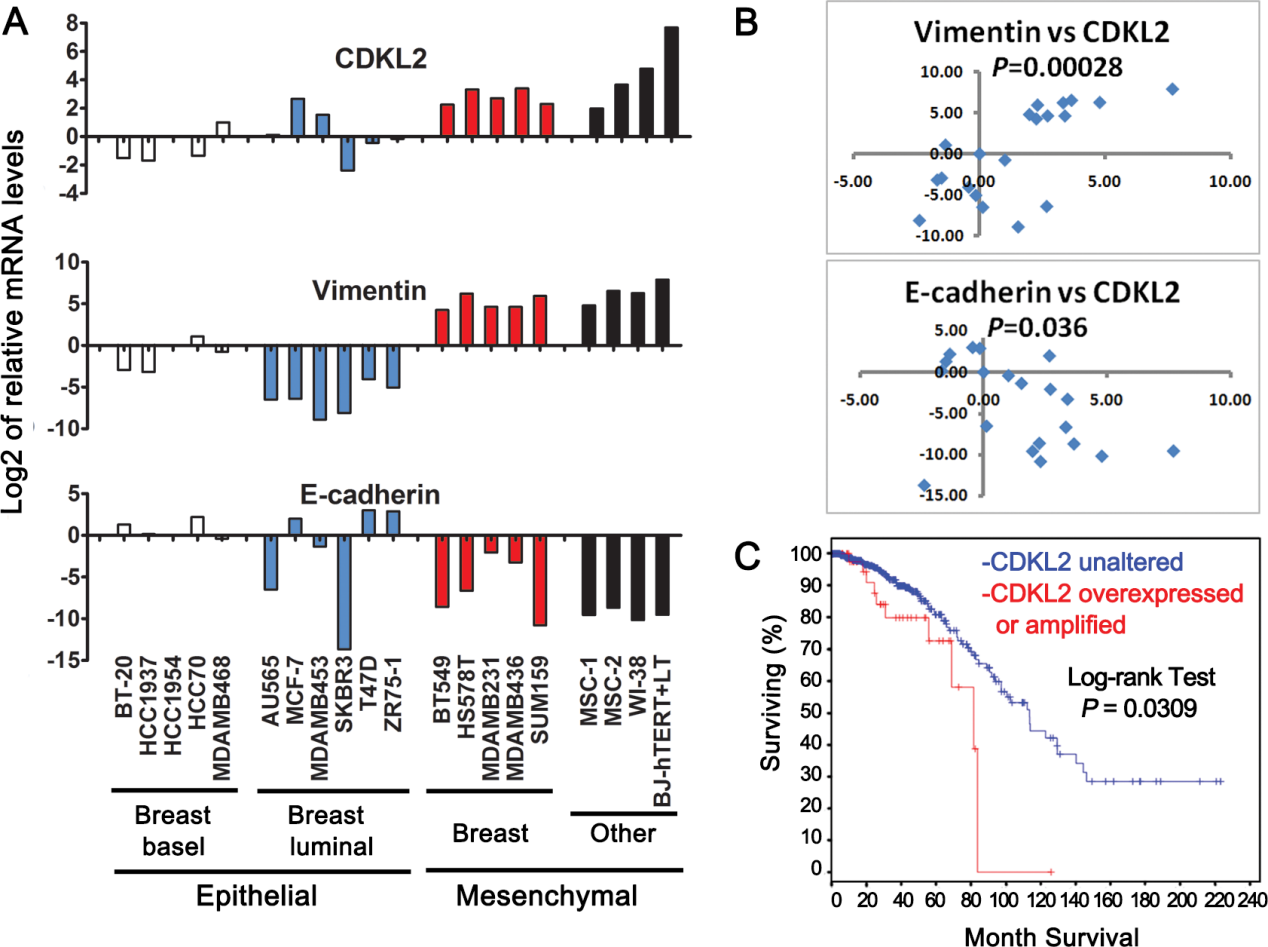
CDKL2 expression in human breast cancer cell lines and human invasive breast cancers **(A)** CDKL2, E-cadherin and Vimentin mRNA levels in a panel of epithelial (basal, luminal) and mesenchymal human breast cancer cell lines, as well as human mesenchymal stem cell (MSC) and fibroblast cell lines. Shown on Y axis is log2 transformation of fold changes for CDKL2, Vimentin and E-cadherin in the indicated cell lines relative to HCC1954 cell line. **(B)** scatter plots of log2 of fold changes for CDKL2 vs Vimentin (top, Spearman correlation coefficient rho= 0.73) and E-cadherin (bottom, rho= - 0.47). **(C)** CDKL2 alterations in human invasive breast cancers. A Kaplan-Meier plot of overall survival corresponding to 749 invasive breast cancers from the publicly available TCGA database is shown for two groups with and without CDKL2 amplifications and/or over-expression, as defined by cBio Portal. The *P*-value was calculated using the Log-rank test.

Our data uncovered that CDKL2 promotes EMT, which suggests that CDKL2 may be expressed at higher levels in mesenchymal cells than in other cell types. To test this hypothesis, we set out to measure its expression in a panel of human breast cancer cell lines, including epithelial (luminal, basal) and mesenchymal subtypes, as well as human mesenchymal stem cell (MSC) and fibroblast cell lines. CDKL2 was indeed expressed significantly higher in mesenchymal breast cancer lines than in epithelial (luminal and basal) breast cancer lines (average fold change = 8.0, *P* = 0.00005, Fig. 7A). Consistent with the subtypes of the breast cancer cells [40–42], E-cadherin expression was significantly lower (with an average fold change of 0.023), while vimentin expression was significantly higher (with an average fold change of 596.1), in mesenchymal breast cancer lines relative to epithelial breast cancer lines (Fig. 7A). Interestingly, two human bone marrow derived mesenchymal stem cells (MSC-1 and -2), human lung fibroblast WI-38 and immortalized human foreskin fibroblast BJ-hTERT+LT also expressed CDKL2 at a much higher level than epithelial breast cancer lines with an average fold change of 26.5 (Fig. 7A). Consistent with our observations that CDKL2 promoted EMT, CDKL2 expression positively correlated with mesenchymal marker vimentin (Spearman correlation coefficient rho = 0.73, *P* = 0.00028), and negatively correlated with expression of epithelial marker E-cadherin (Spearman correlation rho = −0.47, *P* = 0.036, Fig. 7B), strongly supporting a tie between CDKL2 and mesenchymal phenotype in human cells.

Results from our orthotopic xenograft model also indicated that CDKL2 promoted breast cancer progression, which suggests that CDKL2 expression may be higher in human breast cancers with poorer prognostics. Due to lack of high quality CDKL2 antibody suitable for IHC staining on a large collection of human breast cancers specimens, we examined CDKL2 expression in 749 invasive breast cancers that were completed by TCGA [43] for analyses of RNA expression, copy number alteration and mutation (obtained from cBioPortal [44, 45]). Among these patients, those who have CDKL2 amplification and/or elevated expression (~8%) had significantly shorter survival time (log-rank test, *P* = 0.0309, Fig. 7C) compared to the patients without these changes. This result in human samples is consistent with our findings in tissue culture and mouse xenograft model, and further indicates that CDKL2 promotes a malignant phenotype of breast cancer.

## DISCUSSION

In order to systematically identify human kinases that are novel regulators of EMT, we carried out an unbiased human kinase cDNA screen. Among the candidate kinases, CDKL2 was particularly interesting and chosen as the focus of this study, because the EMT phenotypes it induced were the strongest among our kinase candidates, even better than known EMT promoters FYN and MET. The potent role of CDKL2 in EMT was further supported by phenotypic and functional analyses both *in vitro* and *in vivo*. We demonstrated that CDKL2 was able to induce EMT in several human epithelial cell lines. Furthermore, CDKL2 endowed HMLE human mammary gland epithelial cells with stem cell-like characteristics, such as CD44^high^/CD24^low^, mammosphere formation and multilineage differentiation. In orthotopic breast cancer xenograft model, expression of CDKL2 gene significantly increased tumor incidence and spontaneous metastasis.

Mechanistically, we demonstrated that CDKL2 activated a positive feedback loop, consisting of ZEB1/E-cadherin/β-catenin, to induce EMT. CDKL2 induced the expression of ZEB1, a well-established transcriptional suppressor for E-cadherin [37], through increasing ZEB1 promoter activity. As a result, E-cadherin expression was reduced and the epithelial barrier was broken down [38], which led to nuclear translocation of β-catenin, as well as elevated β-catenin/TCF4 transcriptional activity. Activated β-catenin in turn increased ZEB1 promoter activity and transcription [36], resulting in further suppression of E-cadherin expression and continuous activation of the positive feedback loop. Due to this positive feedback loop, it was not surprising to observe the progressive development of EMT with passages. The freshly established HMLE-CDKL2 stable cell lines (in about 4 weeks) acquired partial EMT features, showing increased mesenchymal marker expression (Fig. 1B), unimpaired E-cadherin expression (data not shown) and 40~50% of CD44^high^ subpopulation (Fig. 1D, 3A, 3H). Along with the increased culturing time, HMLE-CDKL2 cells progressively shed their epithelial features as they acquired more mesenchymal phenotypes, with some decrease in E-cadherin expression (Fig. 3E, 4A, 5C) and 70~80% CD44^high^ subpopulation (Fig. 3G, 4D, 5F). A complete EMT phenotype was observed in xenograft primary tumors (Fig. 6C), which may be due to total activation of the positive feedback loop *in vivo* with more passages. We further showed that this positive feedback loop can be broken and the EMT phenotypes can be reversed by down-regulating either β-catenin or ZEB1. As indicated in the above referred literature, each component of this feedback loop has been separately reported [23, 36, 37, 39]. Here we provide an integrated view of this feedback loop and establish CDKL2 as a potent initiator of this loop. Further work is required to determine how CDKL2 biochemically activates this positive feedback loop.

Notably, CDKL2 not only increased the quantity of CD44^high^ subpopulation in HMLE cells, it also rendered the CD44^high^ cells with enhanced EMT and stem cell like characteristics, including abilities of migration, mammosphere formation, and multilineage differentiation. These enhanced abilities could be attributed to higher ZEB1 expression and lower E-cadherin expression in CD44^high^ subpopulation from CDKL2-transduced cells than in CD44^high^ subpopulation from EV-transduced cells (Fig. 3E, 4A and Supplementary Fig. S2B). ZEB1 has been shown to be essential for the conversion from CD44^low^ to CD44^high^ state and also for the maintenance of CD44^high^ stem cell-like activity [23]. Although it is widely accepted that breast cancer stem cells (CSCs) are contained in the CD44^high^ cell compartment, CD44^high^ cells can also constitute heterogeneous cell populations, in which CD44^high^ZEB1^high^ cells enrich with enhanced CSC-like properties [23]. Therefore, CD44^high^ZEB1^high^CDH1^low^, specific markers of the CD44^high^ subpopulation in CDKL2-transduced HMLE cells, can also be regarded as a signature for mammary gland epithelial cells with enhanced EMT and stem cell-like characteristics.

In contrast to the widely accepted CSC model, in which CSCs give rise to non-CSC progeny in a unidirectional manner, several recent studies have demonstrated that non-CSCs can acquire CSC-like activity under certain conditions [23, 46–48]. Our results also supported the theory of bidirectional interconversions between stem-like and non-stem-like state. CD24^high^ non-stem-like cells sorted from HMLE cells can be converted into CD44^high^ stem-like cells by CDKL2-induced EMT. These results also explained the origin of the increased CD44^high^ subpopulation in HMLE-CDKL2 cells. It did not result from expansion of the original small CD44^high^ subpopulation in HMLE cells, because CDKL2 could neither preferentially promote the proliferation of CD44^high^ subpopulation, nor affect the differentiation of stem cell-like CD44^high^ cells into epithelial CD24^high^ cells. Instead, the increased CD44^high^ subpopulation in HMLE-CDKL2 cells was primarily due to a true EMT process (Fig. 3H). Similar results were reported for ectopic expression of the transcription factors Snail, Twist or ZEB1 [13, 23]. Taken together, stem-like cells can be obtained through EMT from more differentiated non-stem-like cells, and therefore, in some cases, CSC hierarchy may not be a rigid unidirectional model but a flexible and interconvertable balance between CSCs and more differentiated cell states [49, 50].

Finally, we observed that CDKL2 is expressed significantly higher in mesenchymal subtypes of breast cancer cells, human MSC and fibroblasts than in epithelial subtypes (luminal and basal) of breast cancer cells, which strongly supports a role of CDKL2 in promoting EMT. Notably, human invasive breast cancers with CDKL2 alterations, either amplification and/or elevated expression, had significantly shorter overall survival time. This result corroborates our findings of CDKL2's roles in EMT and breast cancer progression in cell culture and xenograft models, suggesting that CDKL2 may play a physiological role in human breast cancer development. With the development of individualized breast cancer therapies, new prognostic and predictive biomarkers are required to facilitate clinical decision-making processes. Although preliminary and need to be further validated, our results suggested CDKL2 can be a potential prognostic factor for worse outcome and therapeutic target for human invasive breast cancers.

## METHODS

### Detailed materials and methods are described in Supplementary Information

The kinome cDNA screening was performed by transient transfection of three plasmids of candidate kinase, vimentin promoter-firefly luciferase and TK-renilla luciferase into 293T cells. Thirty six hours after transfection, luciferase activities of vimentin promoter was measured and normalized by readout of renilla luciferase activities using Dual Luciferase Reporter Assay Kit from Promega according to their instructions. The vimentin promoter luciferase vector pGL3-VimPro-Luc plasmid was a gift from Dr. Christine Gilles [12]. Other vector constructs, as well as retro- and lenti-viral preparation are described in the Supplementary Information.

APC-conjugated anti-CD44 (clone G44-26) antibody, PE-conjugated anti-CD24 antibody (clone ML5), and DAPI were obtained from BD Biosciences and used for FACS analysis in accordance with the manufacturer's protocol. Migration ability was tested by Boyden chamber assay. Mammosphere culture was performed as described previously [13] with slight modification, as described in the Supplementary Information. Osteoblastic differentiation and adipogenic differentiation of stem-like cells were detected as described previously [14] and in Supplementary Information. The dependence of cell growth on the growth factors and resistance to cytotoxic agents were tested using AlamarBlue assay.

All mouse procedures were approved by the Animal Care and Use Committees of University of Texas Health Science Center at Houston and performed in accordance with institutional polices. Detailed procedures are described in the Supplementary Information.

Data were presented as mean ± SEM or mean ± SD as indicated, and two-tailed Student's *t-* test was used to compare two groups (*P* < 0.05 was considered significant) unless otherwise indicated.

## SUPPLEMENTARY METHODS AND FIGURES


