# Supplementary material for: CDKL2 promotes epithelial-mesenchymal transition and breast cancer progression
Source: Oncotarget. 2014 Oct 24;5(21):10840–53. doi: 10.18632/oncotarget.2535 (PMC4279414; doi:10.18632/oncotarget.2535)
Supplement: Supplementary file 1 [file oncotarget-05-10840-s001.pdf]

SUPPLEMENTARY METHODS AND FIGURES

Cell culture

The immortalized human mammary gland epithelial cells HMLE and foreskin fibroblast BJ-hTERT+LT were generously provided by Dr. Robert Weinberg [1, 2]. HMLE cells were cultured in DMEM/F12 (1:1) medium (Mediatech) supplemented with 10 µg/ml insulin, 10 ng/ml hEGF, 0.5 µg/ml hydrocortisone, and 1% penicillin-streptomycin. PC3 prostate cancer cells, matching to the PC3 cells from ATCC by finger printing, were kindly provided by Dr. Isaiah Fidler [3]. PC3 and SU86.86 pancreatic cancer cells (ATCC) were cultured in RPMI 1640 media (Mediatech), supplemented with 10% FBS (Gibco) and 1% penicillin-streptomycin (Mediatech). BJ-hTERT+LT and human lung fibroblast WI-38 (ATCC) were cultures in DMEM with 10% FBS. ATCC was also the source for the breast cancer cell lines used in this study, including cells of luminal subtype AU565, HCC202, MCF7, SKBR3, T47D, ZR75-1 and MDAMB453; cells of basal-A subtype HCC1954, HCC1937, BT20, MDAMB468 and HCC70; cells of mesenchymal (claudin-low) subtype BT549, HS578T, MDAMB231 and MDAMB436 [2, 4, 5]. These cells were cultured according to ATCC guidance. Claudin-low SUM159 cells (Asterand) were cultured in F12 + 5% FBS, insulin, and hydrocortisone [2]. Human mesenchymal stem cells, MSC-1 and MSC-2, derived from culture of human bone marrow of two different individuals (Allcells, BM3390 and BM3392), were kindly provided by Dr. Pamela Wenzel at UTHSC-Houston.

Vector constructs, retro- and lenti-viral preparation

Our human kinase cDNA collection in pJP1563 retroviral vector has been previously described [6]. The retroviral pBABE-puro empty vector, or with -Twist, -Snail, -V12 H-Ras were kindly provided by Dr. Robert Weinberg [7, 8]. All shRNA constructs in pLKO.1 lentiviral vector were purchased from Sigma-Aldrich (MISSION®shRNA code: shCDKL2-1, TRCN0000000724; shCDKL2-2, TRCN0000000725; shZEB1-1, TRCN0000017563; shZEB1-2, TRCN0000017564; shZEB1-3, TRCN0000017565; shCTNNB1-1, TRCN0000003846; shCTNNB1-2, TRCN0000010824) and packaged into viral particles in 293T cells for transduction according to their recommendation [9].

Western blotting analysis

Cells were lysed in RIPA buffer supplemented with Complete Mini protease inhibitor cocktail and PhosSTOP phosphatase inhibitor cocktail (Roche Applied Science). Protein concentrations were determined using Pierce BCA protein assay kit (Thermo Scientific). The samples were then separated by SDS-PAGE and transferred to PVDF membrane (Bio-Rad). The membrane was blocked with 5% skim milk in TBST for 1h at room temperature, followed by incubation of a primary antibody overnight at 4°C. Primary antibodies used were as follows: anti-vimentin V9 (Abcam) [10], anti-E-cadherin (BD Bioscience Pharmingen) [11], anti-Fibronectin (BD Bioscience Pharmingen) [12], anti-N-cadherin (BD Bioscience Pharmingen) [13], anti-ZEB1 H102 (Santa Cruz Biotechnology) [14], anti-CD44 IM7 (Santa Cruz Biotechnology) [15], anti-ESRP1/2 (Rockland Immunochemicals) [16], anti-β-catenin (Santa Cruz Biotechnology) [17], anti-occludin (Santa Cruz Biotechnology) [18] and anti-β-actin (Santa Cruz Biotechnology). After washes, the membrane was incubated with HRP-conjugated secondary antibodies (Cell Signaling Technology) for 1h at room temperature. The blots were then detected by Pierce ECL Western Blotting Substrate (Thermo Scientific) on Blue Basic Autoradiography Film (Bioexpress).

Immunofluorescence and immunohistochemistry assay

Cells grown on Lab-Tek II Chamber Slide (Thermo Scientific Nunc) were fixed with 4% paraformaldehyde/PBS for 10 min, and permeabilized with 0.2% Trition-100/PBS for 10 min before blocking with blocking buffer (PBS + 5% BSA + 0.1% Tween-20) for 1 h. Cells were then incubated with primary antibody (diluted 1:200) overnight at 4°C. Primary antibodies used were as follows: anti-E-cadherin (BD Bioscience Pharmingen), anti-β-catenin (Santa Cruz Biotechnology), anti-N-cadherin (BD Bioscience Pharmingen). After washing with PBS, secondary antibodies coupled to Alexa-488 or -594 (Invitrogen) were used in the dark at room temperature for 1 h. Cell nuclei were then visualized with DRAQ5 (Invitrogen). Slides were mounted with SlowFade Gold anti-fade reagent (Invitrogen). Images were capturedwith the Leica TCS SP5 Confocal Microscope and LAS AF software.

For tissue staining, slides with 5-µm sections of formalin-fixed, paraffin-embedded samples were deparaffinized and rehydrated before being subjected to antigen retrieval with 10 mM sodium citrate pH 6.0 for 20 min. Slides were then incubated with Peroxidase Suppressor (Thermo Scientific Pierce), Avidin/Biotin blocking solution (Vector Labs) and non-specific binding blocking buffer (PBS + 5% BSA + 0.1% Tween-20), followed by the corresponding primary antibody (overnight at 4°C) and HRP-conjugated secondary antibody (1 h at room temperature). The immunohistochemistry reaction was developed with

a DAB substrate Kit (Vector Labs) before slides were counterstained with hematoxilin and mounted in Di-N-butyl Phthalate in Xylenesolution (DPX, Sigma-Aldrich).

Reverse transcription and quantitative PCR analysis

RNA was isolated from cells using TRIzol reagent (Invitrogen). cDNA was generated by reverse transcription with iScript cDNA Synthesis Kit (Bio-Rad). Real time PCR was performed using SsoFast EvaGreen Supermix (Bio-Rad) in Bio-Rad CFX96 Real-Time PCR Detection System (Bio-Rad) and PCR products were run on 2% agarose gels. For all RT-PCR analysis, either GAPDH or β-actin mRNA was used to normalize RNA input and expression levels were calculated according to the comparative C_T method ($\Delta\Delta C_T$). Primers sequence used to amplify genes were as follows:

AP1(JUN)-F	5′-CCAAAGGATAGTGCGATGTTT-3′
AP1(JUN)-R	5′-CTGTCCCTCTCCACTGCAAC-3′
CD24-F	5′-TCCAGTGAAACAACAACTGGAA-3′
CD24-R	5′-GGTGGTGGCATTAGTTGGAT-3′
CD44s-F	5′-AGGAGCAGCACTTCAGGAGGTTAC-3′
CD44s-R	5′-ACTGGGGTGGAATGTGTCTTGGTC-3′
CD44v8-9 -F	5′-CAGGTTTGGTGGAAGATTTGG-3′
CD44v8-9 -R	5′-TGTCAGAGTAGAAGTTGTTGGATGG-3′
CDH1-F	5′-GGCCAGGAAATCACATCCTA-3′
CDH1-R	5′-GGCAGTGTCTCTCCAAATCC-3′
CDKL2-F	5′-GGAACCCCTATTTCCTGGAG-3′
CDKL2-R	5′-TCCTTGATTTCAGGCAACCT-3′
CTNNB-F	5′-GCTTTCAGTTGAGCTGACCA-3′
CTNNB-R	5′-CAAGTCCAAGATCAGCAGTCTC-3′
ESRP1-F	5′-ACCAAGCCCTCCGACAGT-3′
ESRP1-R	5′-TGCAGGATTTGCCTGACAT-3′
ESRP2-F	5′-GCTGTTATCCTCCATCTACTCAAAG-3′
ESRP2-R	5′-GTCCACCACATCAGCCTTG-3′
SLUG-F	5′-ACAGCGAACTGGACACACAT-3′
SLUG-R	5′-GATGGGGCTGTATGCTCCT-3′
SNAIL-F	5′-GCTGCAGGACTCTAATCCAGA-3′
SNAIL-R	5′-ATCTCCGGAGGTGGGATG-3′
SOX-2-F	5′-ATGGGTTCGGTGGTCAAGT-3′
SOX-2-R	5′-GGAGGAAGAGGTAACCACAGG-3′
SP1-F	5′-CTATAGCAAATGCCCCAGGT-3′
SP1-R	5′CTGGGCTGTTTTCTCCTTC-3′
TWIST-F	5′-CCCAACTCCCAGACACCTC-3′
TWIST-R	5′-CAAAAAGAAAGCGCCCAAC-3′
VIMENTIN-F	5′-AAAGTGTGGCTGCCAAGAAC-3′
VIMENTIN-R	5′-AGCCTCAGAGAGGTCAGCAA-3′
ZEB1-F	5′-TGTTACCAGGGAGGAGCAGT-3′

ZEB1-R	5′-GCTTCATCTGCCTGAGCTTC-3′
ZEB2-F	5′-ACAAGCCAGGGACAGATCA-3′
ZEB2-R	5′-GCCACACTCTGTGCATTTGA-3′

Migration assay

Cells were starved by culturing in medium without growth factors overnight. After starvation, cells were split and 5×10^4 cells were seeded onto Transwell membrane inserts (24-well transwell plate containing 8 μm pore-size polycarbonate filters, Corning Costar) in growth factors-free medium. Full media was added to the lower chamber. After 24 h incubation at 37°C, non-migrating cells were wiped from the upper side of the membrane. Cells that migrated to the lower surface of the membrane were fixed and stained with 0.5% crystal violet solution (in 95% ethanol). Duplicate inserts were used for each individual experiment, and five random microscopic fields were counted per insert.

Mammoshpere assay

HMLE cells carrying empty vector or expressing EMT regulators were seeded at 5×10^3 cells per well of a 24-well Ultra Low Plate (Corning) and grown for 14 days in DMEM/F12 (1:1) medium (Mediatech) supplemented with 20 ng/ml EGF, 20 ng/ml FGF (Cell Signaling Technology), 4 μg/ml heparin (Sigma), 2% Serum-Free Supplement B27 (Invitrogen) and 1% methylcellulose (Spectrum Chemicals). Wells were fed every 3 days with 0.5 ml media for 2 weeks. Then the mammospheres with diameter > 75 μm were counted at indicated time.

Multilineage differentiation

For osteoblast differentiation, 1×10^4 cells were cultured in NH OsteoDiff Medium (MiltenyiBiotec) in 12-well plates for 35 days; and for adipocyte differentiation, 1×10^5 cells were cultured in NH AdipoDiff Medium (MiltenyiBiotec) for 42 days. The medium was replaced every 3 days. After culturing, committed osteogenic cells and adipogenic cells were detected as previously described [19, 20]. Briefly, to test osteoblastic differentiation, cells were incubated with FAST BCIP/NBT (Sigma-Aldrich) to determine alkaline phosphatase (AP) activity, or analyzed by alizarin red S (Sigma-Aldrich) staining and silver nitrate (Von-Kossa) staining to determine calcium deposition and mineral deposition. As to adipocyte differentiation, oil red O dye (Sigma-Aldrich) staining was used to detect oil droplets formation.

Characterization of cell growth

Cells (300/well) were plated in 96-well plate. After 24 h, cells were gently washed with PBS and cultured with DMEM/F12 (1:1) medium supplemented with decreasing concentrations of growth factors for 12–14 days. Medium with full amounts of growth factors was regarded as 100% growth factors.The medium was replaced every 3 days. Cell proliferation was then measured using the AlamarBlue assay. Briefly, AlamarBlue reagent (invitrogen) was added to each well, and the plate was incubated at 37°C for 6 h. The plates were then read on the Tecan Infinite M1000 plate reader (TECAN Groups Ltd., Mannedorf, Switzerland) using an excitation wavelength of 535nm and an emission wavelength of 595nm. For drug resistance assay, all compounds were purchased from Sigma-Aldrich and dissolved in DMSO. Cells (3000/well) were plated in 100 μl per well in 96-well plates. One day (24 h) after seeding, compounds were added in quadruplicate per concentration for each cell line. Cell viability was measured after 72 h using the AlamarBlue assay.

Promoter reporter luciferaseassay

Cells were transiently transfected with firefly luciferase reporter vectors using TransIT-LT1 Transfection Reagent (MirusBio). As internal control for transfection efficiency, renilla luciferase construct was cotransfected with reporter vectors. After 40 h, luciferase activity was assessed with Dual-Luciferase Reporter Assay System (Promega). All data were at least triplicate and analyzed using Student's t-test.

Animal experiments

NOD/SCID mice were purchased from Charles River Laboratories. All mouse procedures were approved by the Animal Care and Use Committees of University of Texas Health Science Center at Houston and performed in accordance with institutional polices.

HMLER-EV and HMLER-CDKL2 cells were transduced to express luciferase and in vitro luciferase assay was performed to ensure similar luciferase expression in both cell lines. The indicated number of cells were suspended in 30 μl 1:1 Matrigel (BD Bioscience):DMEM/F12 solution and then injected into the 4th inguinal mammary glands on both sides of NOD/SCID mice. Mice were sacrificed after 12 weeks or when tumors reached a diameter > 1 cm.

Tumor size was measured with a slide caliper and the volumes were estimated according to the following formula: Tumor volume (mm^3) = $\pi/6 \times L \times W^2$, where L is length and W is width. For bioluminescence imagining, mice were anaesthetized using ketamin/xylazine and

injected intraperitoneally with 150 mg/kg luciferin (Caliper Life sciences), and then primary tumor and metastasis were imaged using an IVIS Lumina II platform (Caliper Life sciences). Metastasis to lung, liver and chest bones were examined ex vivo upon sacrifice.

SUPPLEMENTARY REFERENCES

1. Elenbaas B, Spirio L, Koerner F, Fleming MD, Zimonjic DB, Donaher JL, Popescu NC, Hahn WC, Weinberg RA. Human breast cancer cells generated by oncogenic transformation of primary mammary epithelial cells. Genes Dev. 2001; 15:50–65.
2. Hahn WC, Counter CM, Lundberg AS, Beijersbergen RL, Brooks MW, Weinberg RA. Creation of human tumour cells with defined genetic elements. Nature. 1999; 400: 464–468.
3. Pettaway CA, Pathak S, Greene G, Ramirez E, Wilson MR, Killion JJ, Fidler IJ. Selection of highly metastatic variants of different human prostatic carcinomas using orthotopic implantation in nude mice. Clin Cancer Res. 1996; 2:1627–1636.
4. Neve RM, Chin K, Fridlyand J, Yeh J, Baehner FL, Fevr T, Clark L, Bayani N, Coppe JP, Tong F, Speed T, Spellman PT, DeVries S, Lapuk A, Wang NJ, Kuo WL, Stilwell JL, Pinkel D, Albertson DG, Waldman FM, McCormick F, Dickson RB, Johnson MD, Lippman M, Ethier S, Gazdar A, Gray JW. A collection of breast cancer cell lines for the study of functionally distinct cancer subtypes. Cancer Cell. 2006; 10:515–527.
5. Prat A, Parker JS, Karginova O, Fan C, Livasy C, Herschkowitz JI, He X, Perou CM. Phenotypic and molecular characterization of the claudin-low intrinsic subtype of breast cancer. Breast Cancer Res. 2010; 12:R68.
6. Pearlberg J, Degot S, Endege W, Park J, Davies J, Gelfand E, Sawyer J, Conery A, Doench J, Li W, Gonzalez L, Boyce FM, Brizuela L, Labaer J, Grueneberg D, Harlow E. Screens using RNAi and cDNA expression as surrogates for genetics in mammalian tissue culture cells. Cold Spring Harb Symp Quant Biol. 2005; 70:449–459.
7. Mani SA, Guo W, Liao MJ, Eaton EN, Ayyanan A, Zhou AY, Brooks M, Reinhard F, Zhang CC, Shipitsin M, Campbell LL, Polyak K, Brisken C, Yang J, Weinberg RA. The epithelial-mesenchymal transition generates cells with properties of stem cells. Cell. 2008; 133:704–715.
8. Yang J, Mani SA, Donaher JL, Ramaswamy S, Itzykson RA, Come C, Savagner P, Gitelman I, Richardson A, Weinberg RA. Twist, a master regulator of morphogenesis, plays an essential role in tumor metastasis. Cell. 2004; 117:927–939.
9. Moffat J, Grueneberg DA, Yang X, Kim SY, Kloepfer AM, Hinkle G, Piqani B, Eisenhaure TM, Luo B, Grenier JK, Carpenter AE, Foo SY, Stewart SA, Stockwell BR, Hacohen N, Hahn WC, Lander ES, Sabatini DM, Root DE. A lentiviral RNAi library for human and mouse genes applied to an arrayed viral high-content screen. Cell. 2006; 124:1283–1298.
10. Wang L, Pawlak EA, Johnson PJ, Belknap JK, Eades S, Stack S, Cousin H, Black SJ. Impact of laminitis on the canonical Wnt signaling pathway in basal epithelial cells of the equine digital laminae. PLoS One. 2013; 8:e56025.
11. Weng Z, Xin M, Pablo L, Grueneberg D, Hagel M, Bain G, Müller T, Papkoff J. Protection against anoikis and down-regulation of cadherin expression by a regulatable beta-catenin protein. J Biol Chem. 2002; 277:18677–18686.
12. Rhee CS, Sen M, Lu D, Wu C, Leoni L, Rubin J, Corr M, Carson DA. Wnt and frizzled receptors as potential targets for immunotherapy in head and neck squamous cell carcinomas. Oncogene. 2002; 21:6598–6605.
13. Nürnberger J, Bacallao RL, Phillips CL. Inversin forms a complex with catenins and N-cadherin in polarized epithelial cells. Mol Biol Cell. 2002; 13:3096–3106.
14. Sánchez-Tilló E, de Barrios O, Siles L, Cuatrecasas M, Castells A, Postigo A. β-catenin/TCF4 complex induces the epithelial-to-mesenchymal transition (EMT)-activator ZEB1 to regulate tumor invasiveness. Proc Natl Acad Sci USA. 2011; 108:19204–19209.
15. Su HT, Weng CC, Hsiao PJ, Chen LH, Kuo TL, Chen YW, Kuo KK, Cheng KH. Stem cell marker nestin is critical for TGF-β1-mediated tumor progression in pancreatic cancer. Mol Cancer Res. 2013; 11:768–79.
16. Warzecha CC, Jiang P, Amirikian K, Dittmar KA, Lu H, Shen S, Guo W, Xing Y, Carstens RP. An ESRP-regulated splicing programme is abrogated during the epithelial-mesenchymal transition. EMBO J. 2010; 29:3286–300.
17. Dao KH, Rotelli MD, Petersen CL, Kaech S, Nelson WD, Yates JE, Hanlon Newell AE, Olson SB, Druker BJ, Bagby GC. FANCL ubiquitinates β-catenin and enhances its nuclear function. Blood. 2012; 120:323–34.
18. Martin TA, Mansel RE, Jiang WG. Loss of occludin leads to the progression of human breast cancer. Int J Mol Med. 2010; 26:723–34.
19. Battula VL, Evans KW, Hollier BG, Shi Y, Marini FC, Ayyanan A, Wang RY, Brisken C, Guerra R, Andreeff M, Mani SA. Epithelial-mesenchymal transition-derived cells exhibit multilineage differentiation potential similar to mesenchymal stem cells. Stem Cells. 2010; 28:1435–45.
20. Koch TG, Heerkens T, Thomsen PD, Betts DH. Isolation of mesenchymal stem cells from equine umbilical cord blood. BMC Biotechnol. 2007; 7:1–9.

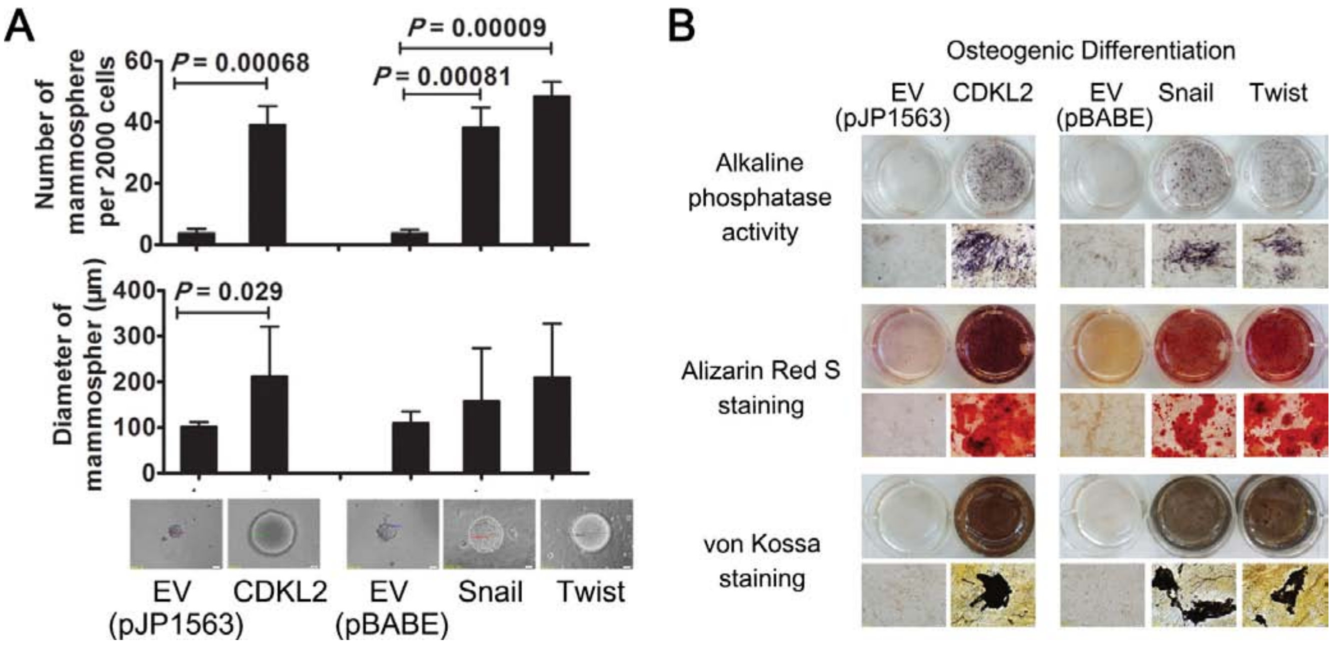

Supplementary Figure S1: The stem cell-like phenotypes induced by CDKL2 is comparable to that induced by Snail and Twist. (A) HMLE-CDKL2 cells generated more mammospheres than HMLE-EV control cells. Snail and Twist were used as positive controls. Phase-contrast images represent mammospheres formed by indicated cell lines. **(B)** HMLE cells expressing CDKL2, Twist or Snail gained MSC-like capabilities for multilineage differentiation. Shown are representative results of osteoblastic differentiation. Following culture in osteoblastic differentiation media, these cells were tested with FAST BCIP/NBT kit to determine alkaline phosphatase (AP) activity, or analyzed by alizarin red S staining and silver nitrate (Von-Kossa) staining to determine calcium deposition and mineral deposition.

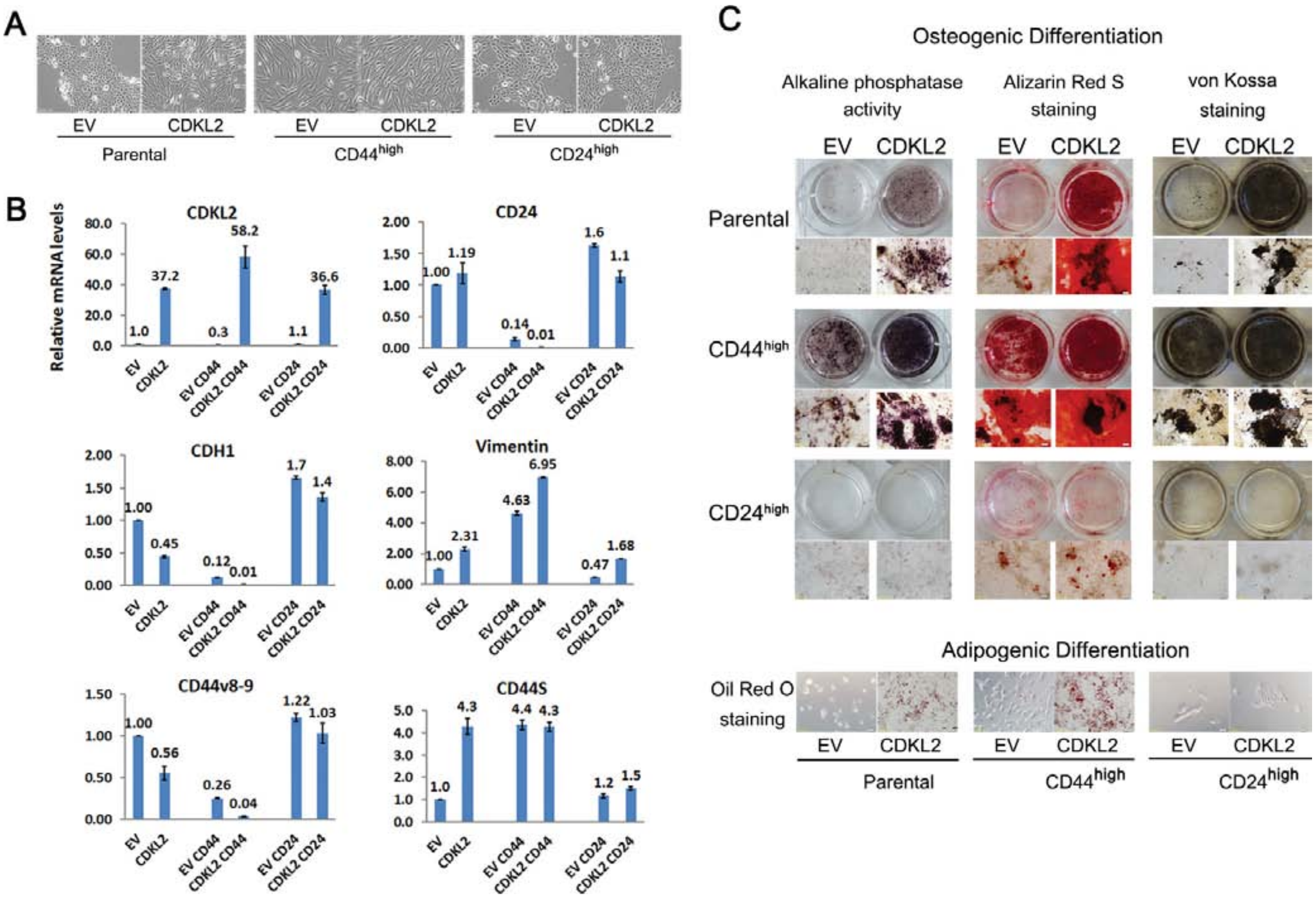

Supplementary Figure S2: CDKL2 endowed CD44^{high}/CD24^{low} subpopulation with enhanced EMT and stem cell-like phenotypes. **(A)** phase-contrast images of parental and sorted cells. **(B)** relative expression of CDKL2, CD24, CD44 splice isoforms (mesenchymal isoform CD44s and epithelial isoform CD44v8-9), and EMT markers (E-cadherin and Vimentin) were measured by real time RT-PCR. β-Actin was used as loading control. The data are reported as mean ± SD. **(C)** multilineage differentiation ability of parental and sorted cells. Following culture in osteoblastic differentiation media, these cells were tested with FAST BCIP/NBT kit to determine alkaline phosphatase (AP) activity, or analyzed by alizarin red S staining and silver nitrate (Von-Kossa) staining to determine calcium deposition and mineral deposition. Following culture in adipogenic differentiation media, cells were stained with oil red dye to detect oil droplets formation.

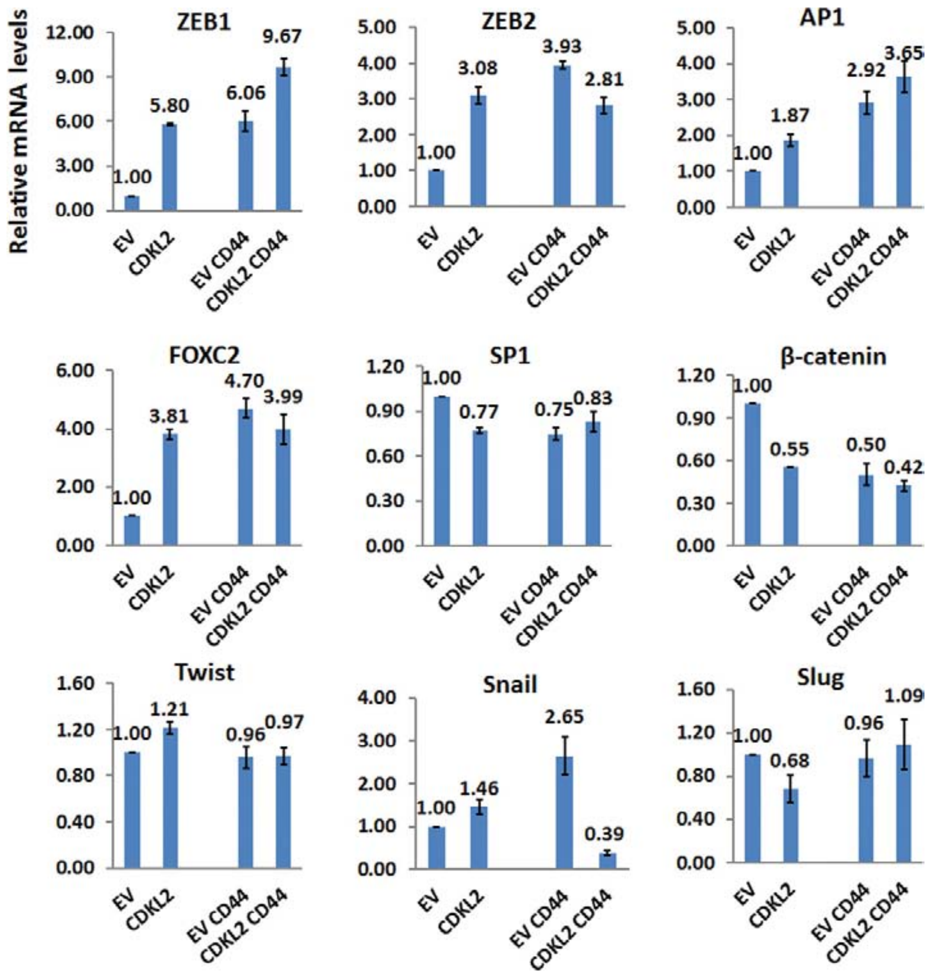

Supplementary Figure S3: Changes of EMT-TFs expression in CDKL2-overexpressing cells. Expression of well-known EMT-TFs (ZEB1, ZEB2, Twist, Snail, Slug, FOXC2, AP1, SP1, and β-catenin) were determined by real time PCR and reported as mean ± SD.

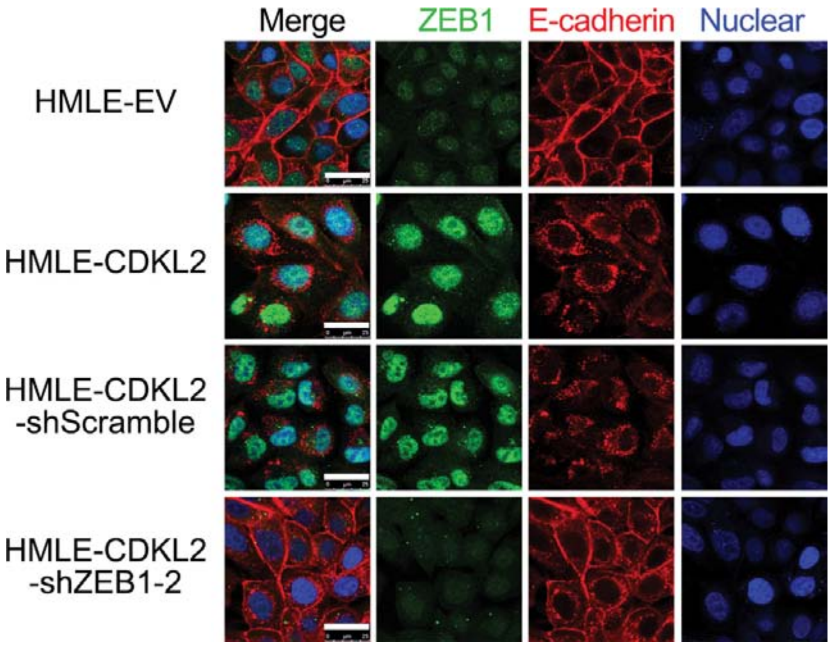

Supplementary Figure S4: Alterations in immunofluorescence staining patterns of ZEB1 and E-cadherin by ZEB1 knockdown in HMLE-CDKL2 cells. Scale bars, 25 µm.
